# Geographic variation in life-history traits: growth season affects age structure, egg size and clutch size in Andrew’s toad (*Bufo andrewsi*)

**DOI:** 10.1186/s12983-016-0138-0

**Published:** 2016-02-09

**Authors:** Wen Bo Liao, Yi Luo, Shang Ling Lou, Di Lu, Robert Jehle

**Affiliations:** Key Laboratory of Southwest China Wildlife Resources Conservation (Ministry of Education), China West Normal University, Nanchong, 637009 Sichuan China; School of Environment & Life Sciences, University of Salford, M5 4WT Salford, UK

**Keywords:** *Bufo andrewsi*, Ectotherms, Environmental variation, Life-history traits, Trade-off

## Abstract

**Background:**

Environmental variation associated with season length is likely to promote differentiation in life-history traits, but has been little studied in natural populations of ectotherms. We investigated patterns of variation in egg size, clutch size, age at sexual maturity, maximum age, mean age, growth rate and adult body size in relation to growth season length among 17 populations of Andrew’s toad (*Bufo andrewsi*) at different latitudes and altitudes in the Hengduan Mountains, western China.

**Results:**

We found that egg size, age at sexual maturity, and mean age increased with decreasing length of the growth season, whereas clutch size showed a converse cline. Body size did not increase with decreasing length of the growth season, but was tightly linked to lifetime activity (i.e. the estimated number of active days during lifetime). Males and females differed in their patterns of geographic variation in growth rates, which may be the result of forces shaping the trade-off between growth and reproduction in different environments.

**Conclusions:**

Our findings suggest that growth season plays an important role in shaping variation in life-history traits in *B. andrewsi* across geographical gradients.

**Electronic supplementary material:**

The online version of this article (doi:10.1186/s12983-016-0138-0) contains supplementary material, which is available to authorized users.

## Background

A central aim in life-history research is to explain patterns of growth, development, reproductive investment and survival at the level of species, populations and individuals [1, 2]. Due to physical and physiological constraints and common dependence on limited resources, investment in one life-history trait is often traded off against investment in other traits [3]. Optimal resource allocation to specific life-history traits for animals across broad environmental ranges depends on differential adaptation to local conditions such as temperature, food supply, predation, and competition, and on the ability for plastic responses [4–7]. Most studies which have examined environmental effects on life-history traits such as age, body size, growth, egg size and clutch size in ectotherms consider that temperature exerts a strong effect [5, 8–18]. For example, many ectotherms delay their reproduction with increasing latitude and/or altitude, while simultaneously decreasing offspring number and increasing offspring size likely as a strategy to enhance offspring survival [10, 16, 19, 20]. Body size is another important life-history trait which has a strong impact on individual fitness, and Bergmann’s rule states that body size and temperature are inversely correlated [21]. Geographic variation in body size predominantly follows Bergmann’s rule in endotherms [22–25], whereas both Bergmann’s rule as well as the reverse of it are frequently reported for ectotherms [14, 26, 27].

In amphibians, latitudinal and/or altitudinal patterns of body size variation may be complex [25]. The influence of temperature on amphibian growth during the larval and juvenile phases affects the timing of maturity and, subsequently, the size of adults [28]. Time constraints associated with the length of the activity season may play an important role in shaping allocation patterns between investment into growth and longevity [26, 29]. For instance, shorter growth seasons should increase the time taken to reach adulthood, leading to a higher age at maturation [8, 30]. This is for example also observed in reptiles, for which longevity often increases with decreasing growth season [7, 31, 32].

*Bufo andrewsi* is a medium-sized anuran with female-biased sexual size dimorphism. The taxonomic validity of *B. andrewsi* as a species distinct from *B. gargarizans* has been debated in recent years [33, 34], but we regard *B. andrewsi* as a distinct species for the present study. *Bufo andrewsi* is widely distributed in subtropical forests of the Hengduan Mountains in China, at elevations ranging from 750 to 3500 m [35]. The species has a relatively long spawning period, with egg laying taking place from early February to mid-June. During the breeding season, males clasp females when they enter the breeding pond, and females produce a single clutch. Egg volume and total reproductive output increase with altitude, whereas clutch size decreases with it [36]. Moreover, in accordance with Bergmann’s rule *B. andrewsi* attain larger adult sizes at metamorphosis, higher average age and larger body sizes despite slower growth rates at higher altitudes [15]. However, differences in average body size among anuran populations not only depend on average age but also on the effective number of days available for growth [16]. Hence, our understanding of observed patterns of interpopulational variation in life-history traits across geographical gradients in amphibians would benefit from further insights into the role of growth season length.

Here, we document the effects of the length of the growth season on geographical variation in life-history traits in *B. andrewsi*. To this end, we analyzed a large dataset derived from 17 distinct populations from the Hengduan Mountains, China. In particular, we investigated how egg size, clutch size, age at maturity, mean age, maximum age and growth rates in males and females vary with variation in the length of the growth season for both sexes. We asked whether differences in effective number of days available for growth can explain the observed variation in body size along geographical gradients.

## Methods

### Sampling of populations

**Fig. 1:**
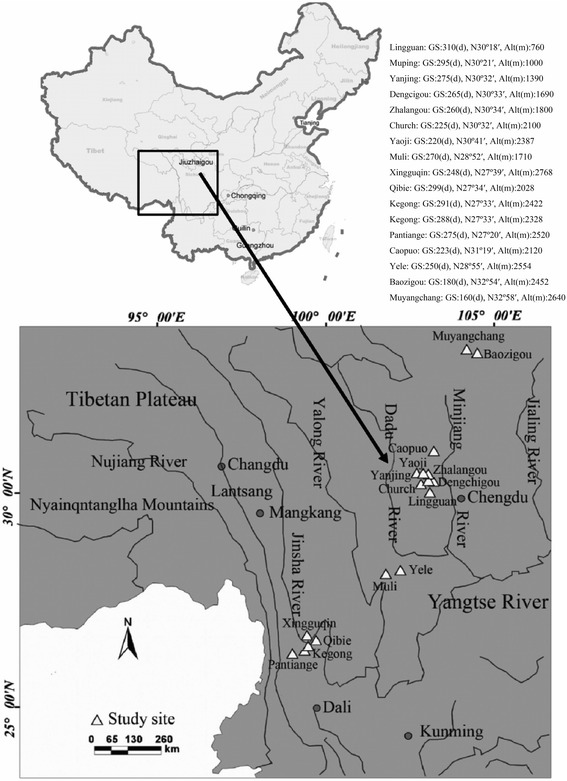
Topographic map showing the location of the 17 *Bufo andrewsi* study populations in the Hengduan Mountains, western China

We sampled a total of 1824 toads (1346 males and 478 females) from 17 populations of *B. andrewsi* between 2007 and 2013 in the Hengduan Mountains, Western China (Fig. 1; Additional file 1: Appendix S1). For all sites, toads were captured by hand on land at night when they were in amplexus or searching for mates. We confirmed all individuals to be adults by direct observation of secondary sexual characteristics (nuptial pads on the first finger for males, eggs readily visible through the skin of the abdomen for females). Only adult individuals migrate to breeding sites, and our data represent the age distribution of the reproductive population [37]. Body size (snout-vent length, SVL, in mm) of each individual was measured to the nearest 0.1 mm using callipers. We surgically removed the second phalange of the longest hind finger and stored it in 4 % neutral buffered formalin for subsequent age determination (see below). After treatment, all individuals were released at the site of their collection.

### Environmental data

Following the protocol of Hjernquist et al. [16], we compiled data on the length of the growth season based on average ambient temperatures between 2007 and 2013 obtained from the Chinese Meteorological Administration (http://www.cma.gov.cn). Field observations revealed that the toads begin to be active when the average daily ambient temperature is approximately 6 °C, a threshold which does not vary among sites (unpublished data); we assume that the length of the growth season closely follows the length of the activity period. In our study area, the length of the growth season (i.e. number of days with a mean daily temperature above 6 °C) differs between localities by a maximum of approximately 150 days (Lingguan and Muyangchang, see Additional file 1: Appendix S1). Low latitude populations in the southern Yunnan province become active in early February, continuing activity until early December. In high latitude populations in the northern Sichuan province, on the other hand, the activity period starts in early May and finishes by mid-October. The length of the growth season can also be used to calculate the lifetime activity period of an individual, by multiplying age (in years) with the length of the growth season in days (following Hjernquist et al. [16]).

### Clutch data

We collected a total of 208 amplectant toads from ten populations (see details in Additional file 1: Appendix S1) between 2008 and 2013, and transported them to laboratories close to spawning sites. We kept pairs separately in tanks (40 × 50 × 60 cm) filled with pond water and allowed them to oviposit. Once oviposition was completed, we counted the total number of eggs in each clutch, and measured SVL of males and females to the nearest 0.1 mm using callipers. We placed 100 randomly selected eggs from each clutch on a glass plate to take a digital image, and measured egg diameter using these photographs (to the nearest 0.01 mm, not including the jelly capsules) at 400× magnification using a Motic Images 3.1 digital camera mounted on a Moticam 2006 light microscope. After the experiments, we returned all individuals and egg strings to their place of collection.

### Age determination

Skeletrochronology is considered the most reliable method of age estimation in wild amphibians, and is based on counting lines of arrested growth (LAGs) in long bones [38, 39]. Paraffin sectioning and Harris’s haematoxylin staining was used to produce histological sections (for details see [40]). The collected digits were washed in water for 2 h and then decalcified in 5 % nitric acid for 48 h, before being washed in running tap water overnight and stained with Ehrlich’s haematoxylin for 75 min. The stained bones were dehydrated and embedded in small paraffin blocks. We selected the cross-section (approximately 13 μm thick) of the phalanx which had the smallest medullar cavity and the thickest cortical bone, and mounted it on glass slides. We recorded the number of lines of arrested growth from mid-diaphyseal sections under a light microscope, having previously confirmed that the number of LAGs reflects actual age based on four marked and recaptured individuals [15]. We assessed endosteal resorption of LAGs by comparing the diameter of the smallest cross-section of 1-year old toads to the diameter of the resorption line in adults [38]. Double lines and false lines could be readily distinguished from the true LAGs and therefore did not create problems for age estimation. In total, we determined the age of 1563 individuals (1166 males and 397 females).

The minimum age of adult toads at a spawning location was used as an estimate of the age at first reproduction in a population, and the maximum age as an estimate of longevity. This is reasonable because the toads captured at the spawning sites represent the age distribution of the reproductive population. We did not find a significant effect of sample size on minimum and maximum age in each population (both *P* > 0.12).

### Growth rates

Von Bertalanffy’s [41] model, a standard method for describing the growth of animals with asymptotic growth after maturity [42–44], was used to estimate growth parameters. The equation has the form of SVL_t_ = SVL_max_ (1 – e^-kt+b^), where SVL_t_ is body size at age t for per individual, SVL_max_ is the estimated asymptotic maximum size in a population, k is a growth coefficient and b is a constant. Growth rate can then be presented as R = dSVL/dt = k (SVL_max_ –SVL_t_).

### Statistical analyses

All analyses were conducted using Type III sums of squares tests in SPSS 21.0 (Statistical Product and Service Solutions Company, Chicago, USA). Differences in minimum age (or mean age or maximum age or lifetime activity) among the 17 populations were tested for using Generalized Linear Mixed Models (GLMMs), with minimum age (or age or maximum age or lifetime activity) as the dependent variable, growth season as fixed effect, and sex, latitude (meters from the equator) and altitude as covariates; population was treated as a random factor. To investigate variation in body size, we treated body size as the dependent variable, growth season as fixed effect, and age, sex, latitude and altitude as covariates, and population as a random factor. To investigate variation in egg size, we treated egg size as the dependent variable, growth season as fixed effect, and female size, latitude and altitude as covariates. Finally, to test variation in egg size and a trade-off between clutch size and egg size, we treated clutch size as the dependent variable, growth season as fixed effect, and female size, latitude, altitude, egg size as covariates.

## Results

### Egg size and clutch size

**Fig. 2:**
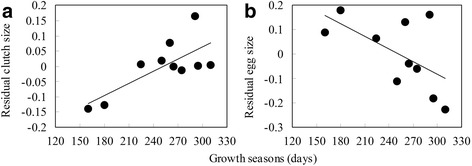
Relationships between length of growth seasons and residual egg size (**a**) as well as residual clutch size (**b**) in *Bufo andrewsi*. Data points are population means. Residuals were generated from regression of egg size or clutch size on female body size. Statistical details are as in Table 1

The GLMM revealed that average egg size was significantly predicted by the length of the growth season (*F*_1,3.852_ = 4.474, *P* < 0.01), and not predicted by latitude, altitude and female body size (Table 1, Fig. 2a). Mean clutch size increased with increasing length of the growth season (*F*_1,5.766_ = 4.474, *P* < 0.05) after controlling for female size, latitude and altitude. Clutch size was positively correlated with female body size (*F*_1,190.801_ = 112.855, *P* < 0.001), but did not increase with latitude and altitude across populations (Table 1; Fig. 2b); however, one population (YL) showed a deviation from the general pattern (Fig. 2). Clutch size was negatively correlated with egg size (Table 1; *F*_1,195.712_ = 6.772, *P* < 0.01). The correlation matrix of population means between the raw variables is shown Additional file 2: Appendix S2.

**Table 1:** Response of egg size (1), clutch size (2), minimum age (3), maximum age (4), average age (5), lifetime activity (6), and adult body length (7) to length of the growth season for 17 *Bufo andrewsi* populations along a geographical gradient as revealed by by General Linear Mixed Models (GLMM)

Source of variation		Random		Fixed	
	Var	SE	Z	df	F
1. Egg size					
Population	0.025	0.021	0.182		
Residual	0.063	0.006	9.759***		
Latitude				1,3.840	0.792
Altitude				1,3.798	0.754
Growth season				1,3.852	4.474**
Female SVL				1,86.430	0.361
2. Clutch size					
Population	417874	259283	1.612		
Residual	288799	29476	9.795***		
Latitude				1,7.769	1.458
Altitude				1,5.739	0.485
Growth season				1,5.766	4.046*
Female SVL				1,190.801	112.855***
Egg size				1,195.712	6.772**
3. Minimum age					
Population	0.157	0.069	2.281*		
Residual	0.108	0.011	10.161***		
Latitude				1,12.954	2.635
Altitude				1,12.856	0.472
Growth season				1,12.796	3.468*
Sex				1,209.987	594.338***
4. Maximum age					
Population	4.353	1.768	2.468*		
Residual	0.435	0.085	5.144***		
Latitude				1,12.908	0.050
Altitude				1,12.895	0.730
Growth season				1,12.908	6.678*
Sex				1.53.275	23.967***
5. Age					
Population	0.945	0.392	2.241*		
Residual	3.089	0.103	30.051***		
Latitude				1,13.779	0.017
Altitude				1,13.835	0.352
Growth season				1,13.692	3.271*
Sex				1,1809.958	184.034***
6. Lifetime activity					
Population	4461	8207	0.524		
Residual	137443	4334	27.668***		
Latitude				1,14.259	0.103
Altitude				1,14.355	0.512
Growth season				1,14.337	0.004
Sex				1,1811.874	176.573***
7. Body size					
Population	45.637	18.133	2.517*		
Residual	30.920	1.029	30.042***		
Latitude				1,13.177	0.370
Altitude				1,13.187	0.068
Growth season				1,13.157	0.479
Sex				1,1806.435	3035.206***
Age				1,1810.021	189.421***

Note: * means significant level. *** *P* < 0.001; **P < 0.01,*P < 0.05

### Age and lifetime activity

**Fig. 3:**
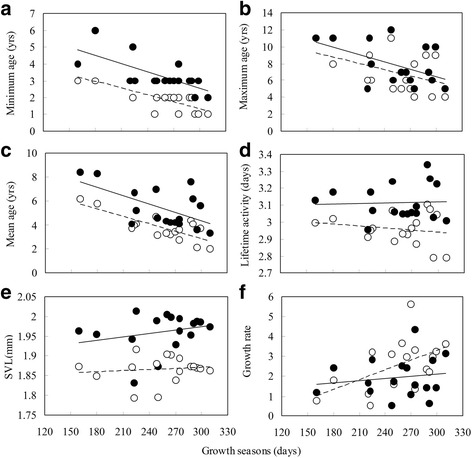
Relationships between length of growth seasons and minimum age (**a**), Maximum age (**b**), mean age (**c**) and lifetime activity (**d**), body size (**e**) and growth rate (**f**) in *Bufo andrewsi*. Solid lines (*filled circles*): females; dashed line (*open circles*): males. Data points are female and male population means. Statistical details are as in Table 1

The GLMMs showed that minimum and maximum age increased with decreasing length of the growth season (minimum age: *F*_1,12.796_ = 3.468, *P* < 0.05; maximum age: *F*_1,12.908_ *=* 6.678, *P* < 0.05). Populations with shorter growth seasons had a higher minimum and maximum age than populations with longer growth seasons (Table 1; Fig. 3a, b), and females had a higher minimum (*F*_1,209.987_ = 594.338, *P* < 0.001) and maximum (*F*_1.53.275_ *=* 23.967, *P* < 0.001) age than males. Similarly, average age increased with decreasing length of the growth season (*F*_1, 13.692_ = 3.271, *P* < 0.05), and average age in females was higher than in males (*F*_1, 1809.958_ = 184.034, *P* < 0.001; Table 1; Fig. 3c). The effects of both altitude and latitude on age structure were non-significant across populations. Lifetime activity was longer in females than in males (*F*_1, 1811.874_ = 176.573, *P* < 0.001), and did not change with length of the growth season, altitude and latitude because populations with longer growth seasons tended to have lower longevity (Table 1; Fig. 3d).

### Body size and growth rate

Although mean body size was not predicted by the length of the growth season, altitude or latitude, the effects of sex and age on body size were significant across populations (Table 1; Fig. 3e; sex: *F*_1,1806.435_ = 3035.206, *P* < 0.001; age: *F*_1,1810.021_ = 189.421, *P* < 0.001). The relationship between growth rate and length of the growth season tended to be sex-specific. Male growth rates increased with decreasing length of the growth season, whereas female growth rate tended to decrease with it (Fig. 3f).

## Discussion

Our analysis of life-history traits across 17 *B. andrewsi* populations provides strong support for the notion that life-history strategies respond to environmental conditions. Specifically, we find that egg size, minimum age at first reproduction, longevity and mean age increase with decreasing length of the growth season, whereas clutch size displays a converse cline. In other words, populations with longer growth seasons mature earlier, have a smaller mean age and longevity, and produce smaller eggs and larger clutch sizes. However, body size and lifetime activity in *B. andrewsi* does not increase with decreasing length of the growth season.

Like in most other ectotherms [7, 45], life-history traits and strategies of anurans are influenced by varying environmental conditions [5, 8, 13–16]. The observed increase in egg size with decreasing length of the growth season may be linked to adaptive beneficial effects on larval and metamorphic performance [10] and, hence, can also influence lifetime fitness [46, 47]. Larger eggs contain a higher amount of yolk [48], which is assumed to be advantageous under the high energetic requirements posed by cold environments. Deviations from the general pattern of egg size by the YL population might relate to differences in temperature regimes between breeding sites, for which no local temperature measurements are available. Maternal condition can influence investment in clutch size [49], and lower resource availability possibly leads to resource limitation on clutch production at shorter activity periods. We observed a trade-off between egg size and clutch size, suggesting investment in larger eggs comes at a cost to egg numbers in accordance with the basic tenets of life-history theory [50].

The larger age at sexual maturity and mean age at shorter growth season in *B. andrewsi* is consistent with previous studies [7, 16, 51]. In populations with longer growth seasons such as Lingguan, Muping and Qibie, toads matured at the age of 1-2 years, and the average age of reproducing toads was 5.1 years. At populations with shorter growth season such as Caopuo, Baozhigou and Muyangchang, individuals matured at the age of 3-4 years, and the mean age of reproducing individuals was 7.1 years. Earlier maturation and lower age in populations with longer growth seasons may reflect higher predation risks [52, 53]. In addition, variation in environmental temperature is likely correlated with fluctuation of food availability. Invertebrates, the major food resources of anurans, are less abundant in low-temperature environments [54]. Low food availability at shorter growth seasons might increase juvenile mortality and juveniles might need longer to reach adulthood, resulting in a higher age at sexual maturity and mean age [45, 49]. In *B. andrewsi*, predation risk and food availability of juveniles and adults are expected to be lower in populations with shorter growth seasons [37]. Thus, temperature-dependent metabolic rates may contribute to the observed variation in sexual maturation and mean age [10, 55]. In addition to minimum and mean age, maximum age in ectotherms often increases with decreasing length of the growth season [7, 16, 55].

As has been observed in previous studies [16, 29], mean body size did not increase with decreasing length of the growth season, despite a strong negative correlation between age at maturity and growth season length. For *B. andrewsi,* lifetime activity therefore cannot explain the observed variation in body size among populations, probably due to a non-significant correlation between the length of the growth season and body size variation. A negative correlation between growth rate and maximum age across geographic gradients has previously been suggested to explain observed body size clines [10]. For instance, later reproduction and higher maximum age at long growth seasons play a more important role in increasing body size in anurans than slower growth does in reducing size, resulting in a negative correlation between body size and growth season [15, 43]. A lifetime growth period, on the other hand, will fail to compensate for the effects of slow growth on body size, resulting in a positive correlation between body size and growth season [14, 27]. In this study, a non-significant variation in body size may be attributed to different contributions of growth rates and age structures in determining body size at varying growth season lengths, in agreement with other studies on life-history traits and seasonality in ectotherms [29, 39].

Growth was more rapid in females compared to males for shorter growth seasons, while males had higher growth rates than females in longer growth seasons (interaction of both sex and lifetime activity: *F*_1,1513.102_ = 2.204, *P* < 0.01; Fig. 3f). A previous study suggested that male *B. andrewsi* mature earlier at lower altitudes and latitudes, also showing a more male-biased operational sex ratio [18]. Therefore, stronger male-male competition at longer growth seasons might also select for accelerated growth in males, whereas more balanced sex ratios and weakened male–male competition at shorter growth seasons may result in slower growth [18, 56]. Life-history theory describes a trade-off between reproductive investment and growth [49]. For female *B. andrewsi*, lower reproductive investment and faster growth are observed in longer growth seasons under warmer conditions [15, 37]. In the present study, we also found a trade-off between reproductive investment and growth through slower growth and higher reproductive investment under environments with shorter activity periods.

Geographical variation in life-history traits in anurans are driven by environmental and genetic differences [5, 26, 57, 58], and plastic responses to environmental conditions are common [26, 59, 60]. Approaches such as common garden experiments are needed to verify the genetic basis for population differences in life-history traits in *B. andrewsi* in the future.

## Conclusions

Taken together, our study shows that individuals from populations with longer seasonal activity periods produce smaller eggs, mature earlier and have larger mean age, whereas individuals with shorter activity periods produce smaller clutches. However, the body sizes do not increase with decreasing length of the growth season across geographical populations. In addition, males and females exhibit differential variation in growth rates across geographical gradients, which can be explained by a trade-off in resource allocation between growth and reproduction in different environments.

## Additional files

Additional file 1: Appendix S1. Descriptive information about the study sites together with minimum age, maximum age, mean body size and age of male and female toads, and clutch size and egg size in female toads. *n* = number of individuals. Descriptive information about the study sites together with mean (± SD) body size, age, egg size and clutch size of toads. *n* = number of individuals. *Data taken from Liao et al. [18]. (DOC 91 kb) Additional file 2: Appendix S2. The correlation matrix of population means between the raw variables in the Andrew’s toad (*Bufo andrewsi*). (DOC 34 kb)

## References

[1] Gadgil M, Bossert W (1971). Life historical consequences of natural selection. Am Nat.

[2] Begon M, Harper JL, Townsend CR (1990). Ecology: individuals, populations and communities.

[3] Roff DA (1992). The evolution of life-histories: theory and analysis.

[4] Sorci G, Clobert J, Belichon S (1996). Phenotypic plasticity of growth and survival in the common lizard *Lacerta vivipara*. J Anim Ecol.

[5] Berven KA (1982). The genetic basis of altitudinal variation in the wood frog *Rana sylvatica*. I. An experimental analysis of life-history traits. Evolution.

[6] Berven KA, Gill DE (1983). Interpreting geographic variation in life-history traits. Am Zool.

[7] Adolph SC, Porter WP (1996). Growth, seasonality and lizard life histories: age and size at maturity. Oikos.

[8] Miaud C, Guyétant R, Elmberg J (1999). Variation in life-history traits in the common frog *Rana temporaria* (Amphibia: Anura): a literature review and new data from the French Alps. J Zool (Lond).

[9] Storz JF, Balasingh J, Bhat HR, Nathan PT, Doss DPS, Prakash AA (2011). Clinal variation in body size and sexual dimorphism in an Indian fruit bat, *Cynopterus sphinx* (Chiroptera: Pteropodidae). Biol J Linn Soc.

[10] Morrison C, Hero JM (2003). Geographic variation in life-history characteristics of amphibians: a review. J Anim Ecol.

[11] Angilletta MJ, Oufiero CE, Leaché AD (2006). Direct and indirect effects of environmental temperature on the evolution of reproductive strategies: an information-theoretic approach. Am Nat.

[12] Gvoždík V, Moravec J, Kratochvíl L (2008). Geographic morphological variation in parapatric Western Palearctic tree frogs, *Hyla arborea* and *Hyla savignyi*: are related species similarly affected by climatic conditions?. Biol J Linn Soc.

[13] Liao WB, Lu X (2010). Age structure and body size of the Chuanxi Tree Frog *Hyla annectans chuanxiensis* from two different elevations in Sichuan (China). Zool Anz.

[14] Sinsch U, Marangoni F, Oromí N, Leskovar C, Sanuy D, Tejedo M (2010). Proximate mechanisms determining size variability in natterjack toads. J Zool (Lond).

[15] Liao WB, Lu X (2012). Adult body size = *f* (initial size + growth rate × age): explaining the proximate cause of Bergman’s cline in a toad along altitudinal gradients. Evol Ecol.

[16] Hjernquist MB, Söderman F, Jönsson KI, Herczeg G, Laurila A, Merilä J (2012). Seasonality determines patterns of growth and age structure over a geographic gradient in an ectothermic vertebrate. Oecologia.

[17] Liao WB (2013). Evolution of sexual size dimorphism in a frog obeys the inverse of Rensch’s rule. Evol Biol.

[18] Liao WB, Liu WC, Merilä J (2015). Andrew meets Rensch: Sexual size dimorphism and the inverse of Rensch’s rule in Andrew’s toad (*Bufo andrewsi*). Oecologia.

[19] Tinkle DW, Wilbur HM, Tilley SG (1970). Evolutionary strategies in lizard reproduction. Evolution.

[20] Räsänen K, Söderman F, Laurila A, Merilä J (2008). Geographic variation in maternal investment: acidity affects egg size and fecundity in *Rana arvalis*. Ecology.

[21] Bergmann C (1847). Über die Verhältnisse der Wärmeökonomie der Thiere zu ihrer Grösse. Gött Stud.

[22] Olalla-Tárraga MA, Rodríguez MÁ (2007). Energy and interspecific body size patterns of amphibian faunas in Europe and North America: anurans follow Bergmann’s rule, urodeles its converse. Glob Ecol Biogeogr.

[23] Meiri S, Dayan T (2003). On the validity of Bergmann’s rule. J Biogeogr.

[24] Ashton KG, Feldman CR (2003). Bergmann’s rule in nonavian reptiles: turtles follow it, lizards and snakes reverse it. Evolution.

[25] Pincheira-Donoso D, Hodgson DJ, Tregenza T (2008). The evolution of body size under environmental gradients in ectotherms: why should Bergmann’s rule apply to lizards?. BMC Evol Biol.

[26] Mousseau TA (1997). Ectotherms follow the converse to Bergmann’s rule. Evolution.

[27] Ma X, Lu X, Merilä J (2009). Altitudinal decline of body size in a Tibetan frog. J Zool (Lond).

[28] Wells KD (2007). The ecology and behaviour of amphibians.

[29] Laugen AT, Laurila A, Jonsson KI, Soderman F, Merilä J (2005). Do common frogs (*Rana temporaria*) follow Bergmann’s rule?. Evol Ecol Res.

[30] Karl I, Fischer K (2009). Altitudinal and environmental variation in lifespan in Copper butterfly *Lycaena tityrus*. Funct Ecol.

[31] Horvátová TH, Cooney CR, Fitze PS, Oksanen TA, Jelić D, Ghira I (2013). Length of activity season drives geographic variation in body size of a widely distributed lizard. Ecol Evol.

[32] Roitberg ES, Kuranova VN, Bulakhova NA, Orlova VF, Eplanova GV, Zinenko OI (2013). Variation of reproductive traits and female body size in the most widely-ranging reptile species: testing the effects of reproductive mode, lineage, and climate. Evol Biol.

[33] Macey JR, Shulte JA, Larson A, Fang Z, Wang Y, Tuniyev BS (1998). Phylogenetic relationships of toads in the *Bufo bufo* species group from the eastern escarpment of the Tibetan Plateau: a case of vicariance and dispersal. Mol Phylogenet Evol.

[34] Frost DR (2013). Amphibian species of the world: an online reference.

[35] Fei L, Ye CY (2001). The colour handbook of amphibians of Sichuan.

[36] Liao WB, Lu X, Jehle R (2014). Altitudinal variation in maternal investment and trade-off between egg size and clutch size in the Andrew’s Toad. J Zool (Lond).

[37] Liao WB (2009). Elevational variation in the life-history of anurans in a subtropics montane forest of Sichuan, southwestern China. PhD thesis.

[38] Castanet J, Smirina E (1990). Introduction to the skeletochronological method in amphibians and reptiles. Ann Sci Nat Zool.

[39] Sinsch U (2015). Review: Skeletochronological assessment of demographic life-history traits in amphibians. Herpetol J.

[40] Li ST, Wu X, Li DY, Lou SL, Mi ZP, Liao WB (2013). Body size variation of Odorous Frog (*Odorrana grahami*) across altitudinal gradients. Herpetol J.

[41] Von Bertalanffy L (1957). Quantitative laws in metabolism and growth. Quar Rev Biol.

[42] Hemelaar AMS (1988). Age, growth and other population characteristics of *Bufo bufo* from different latitudes and altitudes. J Herpetol.

[43] Lu X, Li B, Liang JJ (2006). Comparative demography of a temperate anuran, *Rana chensinensis*, along a relatively fine altitudinal gradient. Can J Zool.

[44] Liao WB, Zhou CQ, Yang ZS, Hu JC, Lu X (2010). Age, size and growth in two populations of the dark spotted frog *Rana nigromaculata* at different altitudes in southwestern China. Herpetol J.

[45] Angilletta MJ, Steury T, Sears M (2004). Temperature, growth rate, and body size in ectotherms: fitting pieces of a life-history puzzle. Integr Comp Biol.

[46] Smith DC (1987). Adult recruitment in chorus frogs: effects of size and date of metamorphosis. Ecology.

[47] Berven KA (1990). Factors affecting population fluctuations in larval and adult stages of the wood frog (*Rana sylvatica*). Ecology.

[48] Komoroski MJ, Nagle RD, Congdon JD (1998). Relationships of lipids to ovum size in amphibians. Phys Zool.

[49] Gliwicz ZM, Guisande C (1992). Family-planning in Daphnia: resistance to starvation in offspring born to mothers grown at different food levels. Oecologia.

[50] Roff DA (2002). Life-history evolution.

[51] Cvetković D, Tomaśević N, Ficetola GF, Crnobrnja-Isailović J, Miaud C (2009). Bergmann’s rule in amphibians: combining demographic and ecological parameters to explain body size variation among populations in the common toad *Bufo buf*o. J Zool Syst Evol Res.

[52] Charlesworth B (1994). Evolution in age-structured populations.

[53] Schemske DW, Mittelbach GC, Cornell HW, Sobel JM, Kaustuv R (2009). Is there a latitudinal gradient in the importance of biotic interactions?. Ann Rev Ecol Evol Syst.

[54] Lessard JP, Sackett TE, Reynolds WN, Fowler DA, Sanders NJ (2011). Determinants of the detrital arthropod community structure: the effects of temperature, resources, and environmental gradients. Oikos.

[55] Brown JH, Gillooly JF, Allen AP, van Savage M, West GB (2004). Toward a metabolic theory of ecology. Ecology.

[56] Liao WB, Lu X (2011). Proximate mechanisms leading to large male-mating advantage in the Andrew’s toad *Bufo andrewsi*. Behaviour.

[57] Laugen AT, Laurila A, Merilä J (2003). Latitudinal and temperature dependent variation in embryonic development and growth in *Rana temporaria*. Oecologia.

[58] Lindgren B, Laurila A (2005). Proximate causes of adaptive growth rates: growth efficiency variation among latitudinal populations of *Rana temporaria*. J Evol Biol.

[59] Alho JS, Herczeg G, Laugen AT, Räsänen K, Laurila A, Merilä J (2011). Allen’s rule revisited: quantitative genetics of extremity length in the common frog along a latitudinal gradient. J Evol Biol.

[60] Muir AP, Biek R, Thomas R, Mable BK (2014). Local adaptation with high gene flow: temperature parameters drive adaptation to altitude in the common frog (*Rana temporaria*). Mol Ecol.

