# Supplementary material for: Geographic variation in life-history traits: growth season affects age structure, egg size and clutch size in Andrew’s toad (Bufo andrewsi)
Source: Front Zool. 2016 Feb 9;13:6. doi: 10.1186/s12983-016-0138-0 (PMC4748633; doi:10.1186/s12983-016-0138-0)
Supplement: Additional file 1: Appendix S1. — Descriptive information about the study sites together with minimum age, maximum age, mean body size and age of male and female toads, and clutch size and egg size in female toads. n = number of individuals. Descriptive information about the study sites together with mean (± SD) body size, age, egg size and clutch size of toads. n = number of individuals. *Data taken from Liao et al. [18]. (DOC 91 kb) [file 12983_2016_138_MOESM1_ESM.doc]

| Study sites | Altitude  (m) | Latitude (N°) | Length of growth season | Female  SVL(mm) | Male  SVL(mm) | Female  age(yr) | Male  age(yr) | Female  minimum age | Male  minimum age | Female  maximum age | Male maximum age | Egg size | Clutch size |
| --- | --- | --- | --- | --- | --- | --- | --- | --- | --- | --- | --- | --- | --- |
| Lingguan | 760 | 30º18′ | 310 | 94.2  n = 32 | 72.9  n = 47 | 3.3  n = 32 | 2  n = 47 | 2  n = 6 | 1  n =14 | 5  n = 4 | 4  n = 2 | 1.97  n = 21 | 3440  n = 21 |
| Muping | 1000 | 30º21′ | 295 | 97.3  n = 26 | 74.1  n = 46 | 3.6  n = 26 | 2.1  n = 44 | 2  n=1 | 1  n=9 | 6  n=1 | 4  n=2 | 2.05  n = 27 | 3574.6  n = 27 |
| Yanjing | 1390 | 30º32′ | 275 | 98.8  n = 8 | 78.2  n = 18 | 4.1  n = 7 | 2.7  n = 15 | 3  n =2 | 2  n =7 | 5  n =2 | 4  n =3 | 2.19  n = 11 | 3512.7  n = 11 |
| Dengcigou | 1690 | 30º33′ | 265 | 99.7  n = 192 | 79.9  n = 361 | 4.2  n= 120 | 3.2  n = 229 | 3  n = 37 | 2  n = 44 | 7  n = 5 | 6  n =3 | 2.22  n = 41 | 3655.1  n = 41 |
| Zhalangou | 1800 | 30º34′ | 260 | 101.0  n = 9 | 80.2  n = 29 | 4.3  n = 9 | 3.3  n = 11 | 3  n = 3 | 2  n = 2 | 7  n = 1 | 5  n = 2 | 2.22  n = 7 | 3558.7  n = 7 |
| Church | 2100 | 30º32′ | 225 | 103.2  n = 22 | 82.6  n = 30 | 5.3  n = 22 | 4.1  n = 22 | 3  n = 2 | 3  n = 6 | 8  n = 1 | 6  n = 2 | 2.36  n = 21 | 3887.2  n = 21 |
| Yaoji | 2387 | 30º41′ | 220 | 87.5  n = 7 | 74.3  =17 | 4.1  n = 7 | 3.7  n = 15 | 3  n = 2 | 3  n = 7 | 10  n = 1 | 9  n = 2 |  |  |
| Muli | 1710 | 28º52′ | 270 | 84.9  n = 5 | 69.0  n = 12 | 4.2  n = 5 | 3.4  n = 12 | 3  n = 2 | 2  n = 2 | 6  n = 1 | 5  n = 3 |  |  |
| Xingguqin | 2768 | 27º39′ | 248 | 97.4  n = 15 | 76.0  n = 203 | 7.0  n = 14 | 4.7  n = 197 | 3  n = 1 | 1  n = 6 | 12  n = 1 | 11  n = 1 |  |  |
| Qibie | 2028 | 27º34′ | 299 | 96.8  n = 27 | 73.7  n = 167 | 5.6  n = 27 | 3.7  n = 167 | 3  n = 5 | 1  n = 2 | 10  n = 1 | 9  n = 1 |  |  |
| Kegong | 2422 | 27º33′ | 291 | 95.9  n = 17 | 74.8  n = 67 | 6.2  n = 17 | 4.1  n = 67 | 3  n = 4 | 2  n = 7 | 7  n = 2 | 7  n = 1 | 2.38  n = 5 | 5086.6  n = 5 |
| Kegong | 2328 | 27º33′ | 288 | 89.8  n = 4 | 74.6  n = 110 | 7.6  n = 4 | 4.4  n = 110 | 3  n = 1 | 2  n = 9 | 10  n = 1 | 9  n = 2 |  |  |
| Pantiange | 2520 | 27º20′ | 275 | 91.9  n = 13 | 72.6  n = 75 | 4.5  n = 13 | 3.6  n = 75 | 4  n = 5 | 1  n = 2 | 5  n = 6 | 8  n = 1 |  |  |
| Caopuo | 2120 | 31º19′ | 223 | 67.9  n = 9 | 62.2  n = 35 | 4.0  n = 7 | 6.7  n = 25 | 5  n = 2 | 2  n = 6 | 11  n = 1 | 9  n = 1 |  |  |
| Yele | 2554 | 28º55′ | 250 | 74.6  n = 43 | 62.5  n = 63 | 4.6  n = 43 | 3.1  n = 63 | 3  n = 4 | 2  n = 21 | 6  n = 3 | 5  n = 4 | 1.84  n = 26 | 2648.3  n = 26 |
| Baozigou | 2452 | 32º54′ | 180 | 91.0  n = 18 | 74.8  n = 28 | 8.3  n = 18 | 5.8  n = 28 | 6  n = 1 | 3  n = 2 | 11  n = 1 | 8  n = 2 | 2.33  n = 18 | 2389.8  n = 18 |
| Muyangchang | 2640 | 32º58′ | 160 | 91.9  n = 27 | 70.6  n = 47 | 8.4  n = 27 | 6.2  n = 47 | 4  n = 1 | 3  n = 1 | 11  n = 5 | 11  n = 1 | 2.26  n = 27 | 2389.3  n = 27 |

Table S1. Descriptive information about the study sites together with minimum age, maximum age, mean body size and age of male and female toads, and clutch size and egg size in female toads. n = number of individuals.
