# Supplementary material for: Geographic variation in life-history traits: growth season affects age structure, egg size and clutch size in Andrew’s toad (Bufo andrewsi)
Source: Front Zool. 2016 Feb 9;13:6. doi: 10.1186/s12983-016-0138-0 (PMC4748633; doi:10.1186/s12983-016-0138-0)
Supplement: Additional file 2: Appendix S2. — The correlation matrix of population means between the raw variables in the Andrew’s toad (Bufo andrewsi). (DOC 34 kb) [file 12983_2016_138_MOESM2_ESM.doc]

| Variables | Egg size | Clutch size | Female size | Altitude | Latitude | Growth season |
| --- | --- | --- | --- | --- | --- | --- |
| Egg size |  | -1545.876* | 28.764 | 1420.210 | 2.060 | -111.098** |
| Clutch size | -0.001* |  | 0.005* | -0.197 | -0.001* | 0.041* |
| Female size | 0.014 | 52.756* |  | -34.818 | 0.023 | 1.148 |
| Altitude | 0.001 | -0.286 | -0.005 |  | 0.001 | -0.053* |
| Latitude | 0.025 | -375.8* | 0.581 | 39.137 |  | -22.838** |
| Growth season | -0.010** | 10.836* | 0.031 | -9.805* | -0.042** |  |

Table S2. The correlation matrix of population means between the raw variables in the Andrew’s toad (*Bufo andrewsi*)

Note: *P < 0.05; ***P* < 0.01;****P* < 0.001
